# TGF-β promotes the proliferation and osteogenic differentiation of dental pulp stem cells a systematic review and meta-analysis

**DOI:** 10.1186/s40001-023-01227-y

**Published:** 2023-07-27

**Authors:** Pengfei Gao, Chanjuan Liu, Hui Dong, Qi Li, Yunfang Chen

**Affiliations:** 1 Department of Stomatology, Bengbu Medical College, 2600 Donghai Avenue, Bengbu, 233030 China; 2 Center for Plastic & Reconstructive Surgery, Department of Stomatology, Zhejiang Provincial People’s Hospital (Affiliated People’s Hospital), Hangzhou Medical College, Hangzhou, Zhejiang, 310014, China

**Keywords:** TGF-β, Dental pulp stem cells, Osteogenesis, Proliferation

## Abstract

**Background:**

Dental pulp stem cells (DPSCs) are adult stem cells with multi-directional differentiation potential derived from ectoderm. Vitro experiments have shown that adding cytokines can help DPSCs to be transformed from multipotent stem cells to osteoblasts. TGF-β has been proved to have an effect on the proliferation and mineralization of bone tissue, but its effect on the osteogenesis and proliferation of dental pulp stem cells is still uncertain. We aim to determine the effect of TGF-β on the osteogenesis and proliferation of dental pulp stem cells.

**Methods:**

We have identified studies from the Cochrane Central Register of Controlled Trials, PubMed, Embase, and China national knowledge infrastructure (CNKI) for studies interested in TGF-β and proliferation and differentiation of dental pulp stem cells in the following indicators: A490 (an index for evaluating cell proliferation), bone sialoprotein (BSP), Col plasmid-1 (Col-1), osteocalcin (OCN), runt-related transcription factor 2 (Runx-2); and the number of mineralized nodules. Any language restrictions were rejected. Furthermore, we drew a forest plot for each outcome. We conducted a sensitivity analysis, data analysis, heterogeneity, and publication bias test. We evaluate the quality of each study under the guidance of Cochrane's tool for quality assessment.

**Results:**

The pooled data showed that TGF-β could promote the proliferation and ossification of dental pulp stem cells. All the included results support this conclusion except for the number of mineralized nodules: TGF-β increases the A490 index (SMD 3.11, 95% CI [0.54–5.69]), promotes the production of BSP (SMD 3.11, 95% CI [0.81–6.77]), promotes the expression of Col-1 (SMD 4.71, 95% CI [1.25–8.16]) and Runx-2 (SMD 3.37, 95% CI [− 0.63 to 7.36]), increases the content of OCN (SMD 4.32, 95% CI [1.20–7.44]) in dental pulp, and has no significant effect on the number of mineralized nodules (SMD 3.87, 95% CI [− 1.76 to 9.51]) in dental pulp stem cells.

**Conclusions:**

TGF-β promotes the proliferation and osteogenesis of dental pulp stem cells.

**Supplementary Information:**

The online version contains supplementary material available at 10.1186/s40001-023-01227-y.

## Introduction

The transforming growth factor-&beta; (TGF-&beta/TGF-β) subfamily mainly includes three isoforms of TGF-β1–3, which have similar biological activity and high homology in amino acid sequence [1], and TGF-β is a cytokine that is secreted by various cell types, including macrophages, and functions in cell growth, cell differentiation, apoptosis and cellular homeostasis by binding to its receptor, the TGFβ type II receptor. TGF-β mRNA and its protein were expressed in different stages of tooth germ formation, including early bud stage and root dentin formation. TGF superfamily growth factor mediates transduction signals through its corresponding receptors [2]. For example, the TGF-β, the classical signal transduction pathway, is mediated by Sma and Mad proteins (Smad protein) and transduced by TGF-β1 receptor (TGF-βR1) and TGF-βR2 [3]. Dental pulp stem cells (DPSCs) are adult stem cells with multi-directional differentiation potential derived from ectoderm. Proliferation and differentiation of DPSCs and up-regulation of various growth factors were observed during tooth development and pulp repair [4]. Bone sialoprotein (BSP) is an acidic glycoprotein in the extracellular matrix, and its tissue distribution is relatively limited [5]. It is mainly distributed in mineralized tissues (such as bone and teeth) and calcified cartilage and bone junction. Its content accounts for a lot of the non-glial protein in the bone extracellular matrix. The BSP is the indicator for the proliferation of dental pulp. Osteoblasts and odontoblasts mainly synthesize osteocalcin, and proliferative chondrocytes synthesize some. It plays an essential role in regulating bone calcium metabolism. It is a new biochemical marker for studying bone metabolism [6], which is used in studies as an indicator to assess the mineralization of dental pulp. Runt-related transcription factor 2 is an essential protein for osteoblast differentiation, which we use to evaluate the proliferation of dental pulp stem cells.

Vitro experiments have shown that adding cytokines can help DPSCs to be transformed from multipotent stem cells to osteoblasts [7]. This transform may have potential clinical value in dental fields, such as tooth transplantation or tooth regeneration. More and more researchers have supported the critical role of TGF-β in promoting cell growth and differentiation, including its effect on the proliferation and differentiation of dental pulp stem cells [8–10]. Many animal experiments or trials on the differentiation of human dental pulp stem cells and TGF-β have been carried out, and conclusions are quite different [11–23]. The purpose of this study was to summarize the results of the studies above and determine whether TGF-β promotes the proliferation and osteogenic differentiation of dental pulp stem cells.

## Methods

### Literature search

**Fig. 1:**
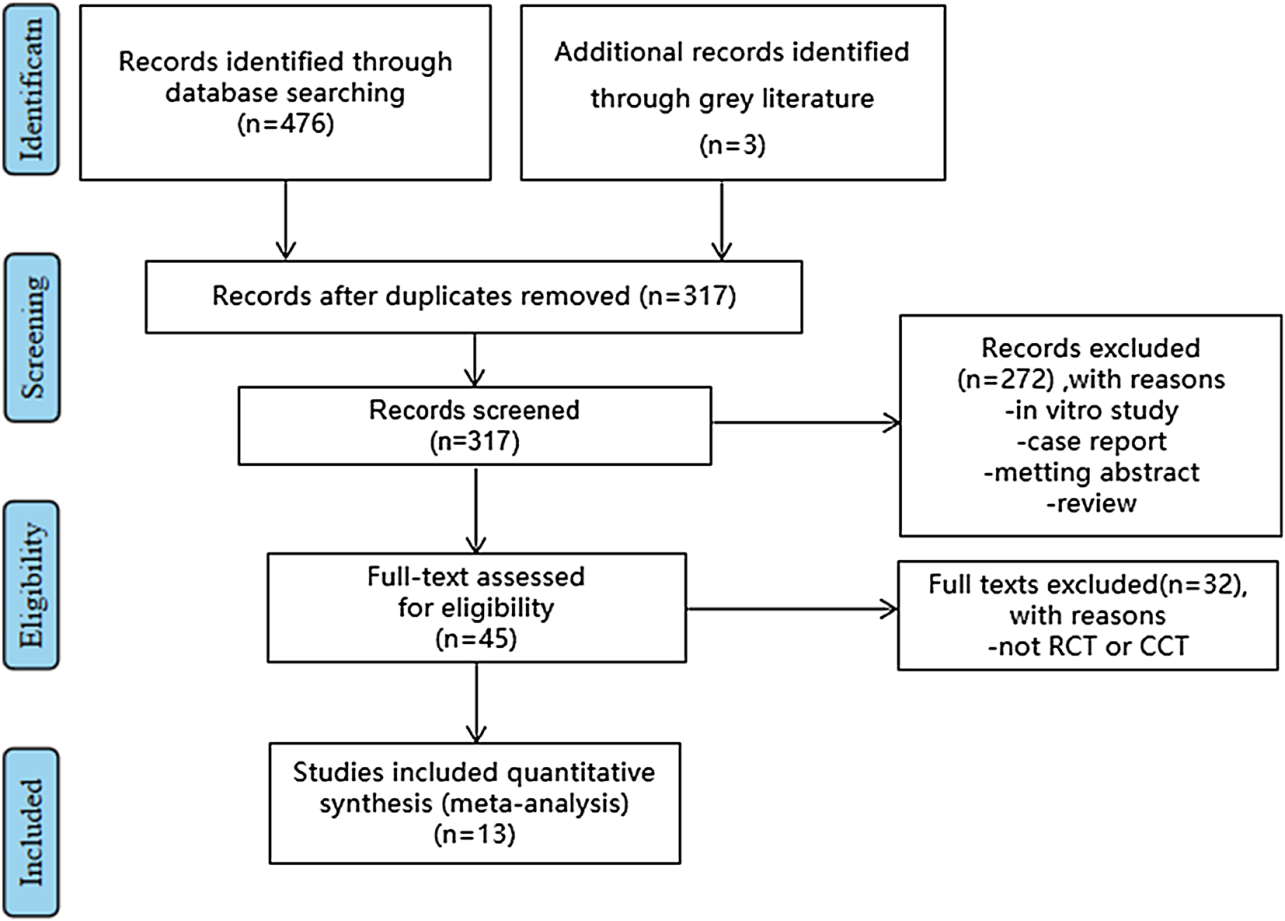
Flow chart of study identifying

We have identified studies from the Cochrane Central Register of Controlled Trials, PubMed, Embase, and CNKI (from August 12, 2012 to September 31, 2021) for studies interested in TGF-β and proliferation and differentiation of dental pulp stem cells in the following indicators: A490 (an index for evaluating cell proliferation), bone sialoprotein (BSP), Col plasmid-1(Col-1), osteocalcin (OCN), runt-related transcription factor 2 (Runx-2); and the number of mineralized nodules. Any language restrictions were rejected. We included both animal experiments and trials on samples from human teeth. These studies were excluded under the following circumstances: This study is not a clinical randomized controlled trial. 2. Research objects or results and interventions do not meet the inclusion criteria. 3. Sample from people or animals with teeth diseases, which may cause uncertainty of outcomes. 4. The incomplete data or full text cannot be obtained. 5. Any conference papers, reviews, case reports, and lessons learned, repetitive literature (multilingual papers retain only the earliest literature) will also be rejected. Moreover, we excluded those indicators and outcomes with insufficient sample size or literature number. We provided a flow chart for the progression of literature search and inclusion or exclusion of studies in Fig. 1. The review was reported according with PRISMA guidelines (Additional file 1: PRISMA Checklist).

### Data analysis

We summarized the effect of TGF-β on proliferation and differentiation of dental pulp stem cells in the following indicators: A490 (an index for evaluating cell proliferation), bone sialoprotein (BSP), Col plasmid-1(Col-1), osteocalcin (OCN), Runt-related transcription factor 2 (Runx-2); and the number of mineralized nodules. Furthermore, we drew a forest plot by Stata16.1 software for each outcome. We conducted a sensitivity analysis, data statistics, heterogeneity, and publication bias test by STATA version 16.1 (STATA 2020). After discussion, we set the critical value of I-square as 45%. When the heterogeneity is more significant than this value, we believe that the consistency of the sample is poor. We performed Begg's test and egger's test to check the publication bias. Quality assessments of each study included were performed by Cochrane's tool for quality assessments of random-controlled trials [24].

### Quality assessment and data extractions

**Fig. 2:**
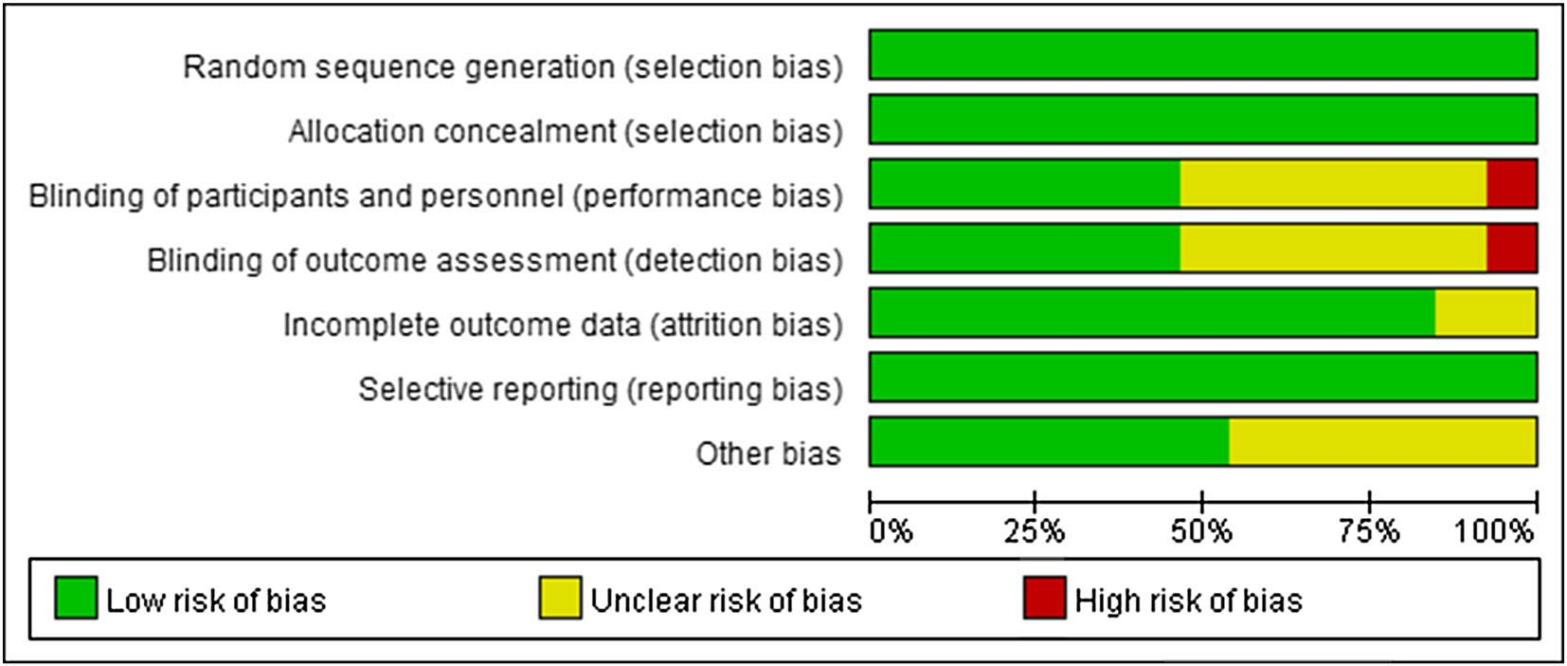
Risk bias

We used Revman 5.4.1 (Revman 2020) software to evaluate the quality of each study under the guidance of Cochrane's tool for quality assessment: 1. Random sequence generation 2. Allocation concealment 3. Blinding of participants and personnel 4. Blinding of outcome assessment 5. Incomplete outcome data 6. Selective reporting 7. Other bias. Results are provided in Fig. 2. Three reviewers (Pengfei Gao, Qi Li, Hui Dong) independently recorded the TGF type, animal species and strains, animal age, and body weight, dose frequency, type of the control group, outcome measures, course of treatment, interventions, adverse reactions, and other characteristics of the included studies, which we summarized in Table 1: Research characteristics.

**Table 1:** Characteristics of studies

Study	Years	Type	Species	Age or weight	TGFdose	Control group	Outcomes	Time/d
Ermeldan Enewal	2019	β3	Rabbit	3–4 years, 1.5–2.0 kg	80 ng/ml	rDPSCs	①③④⑤⑥	7d
Liu	2016	β3	Rabbit	2-3 years, 0.8–1.0 kg	20 ng/ml	DPSCs	①	/
Mai Mai Tiemin Harik1	2019	β3	Rabbit	2-3 years, 0.8–1.0 kg	/	DPSCs	①③⑤	7d
Mai Mai Tiemin Harik2	2016	β3	Rabbit	/	20 ng/ml	DPSCs	①	7d
Wang	2017	β3	Rabbit	4 years, 1–1.2 kg	80 ng/ml	DPSCs	②③④	4-8W
Wu	2015	β3	Rabbit	4-6 years, 0.8–1.2 kg	250 ng/ul	DPSCs	②③④	6W
Han	2017	β3	Rabbit	1–2 years, 0.5–0.8 kg	80 ng/ml	DPSCs	①②	7d
YILIN WANG	2016	β3	Rabbit	4 years, 0.4–0.5 kg	/	DPSCs	①	7d
Guzalinur Ababakli	2016	β3	Rabbit	2 years, 2.5–3.0 kg	80 ng/ml	DPSCs	①④⑤⑥	2-12W
Jia	2017	β1	Human	18-25 years	6 μg/L	DPSCs	①	7d
Ren	2014	β3	Human	7–8 years	25 ng/mL	DPSCs	①②	7d
Xu	2014	β1	Human	/	5 ng/ml	DPSCs	①	7d
Jiang	2018	β1	Human	/	20 ng/mL	DPSCs	②③⑤	7d

① A490; ② BSP;③ OCN;④ COL-1;⑤ Runx-2;⑥ mineralized nodules

## Result

### Literature search

We have identified a total of 479 studies interested TGF-β and proliferation and differentiation of dental pulp stem cells, from which we finally included a total of 13 studies with animal or human dental pulp cells samples from August 12, 2012 to September 31, 2021, on published papers or websites. These studies were excluded under the following circumstances: This study is not a randomized controlled trial. 2. Research objects or results and interventions do not meet the inclusion criteria. 3. Sample from people or animals with teeth diseases, which may cause uncertainty of measures. 4. The complete data or full text cannot be obtained. 5. Any conference papers, reviews, case reports, and lessons learned, repetitive literature (multilingual papers retain only the earliest literature) will also be rejected. In addition, we excluded those indicators and outcomes with insufficient sample size or literature number. We finalized the following six indicators as the outcomes of this systematic review and meta-analysis: A490 (an index for evaluating cell proliferation), bone sialoprotein (BSP), Col plasmid-1 (Col-1), osteocalcin (OCN), Runt-related transcription factor 2 (Runx-2); and the number of mineralized nodules.

### Characteristics of studies

Three reviewers (Pengfei Gao, Qi Li, Hui Dong) independently recorded the TGF type, animal species and strains, animal age, and body weight, dose frequency, control group type, outcomes, course of treatment, interventions, adverse reactions, and other characteristics of the included studies, which we summarized in Table 1: Research characteristics. The results showed that most samples were from children aged 2–4 years or animals weighing 1–3 kg, mainly rabbits. What is more, the observation period is 7 days in most studies. The main types of TGF are β3 and β1. For more details, see Table 1: Research characteristics.

### Data analysis

The TGF types were mainly TGF-β-1, TGF-β-3, except for TGF-β2. The included studies were all randomized controlled trials of samples from animals or people. Furthermore, publication bias has not existed in any outcome included. The heterogeneity of most results is high, suggesting that the evidence we provide is of medium quality and credibility.

#### A 490 Index

**Fig. 3:**
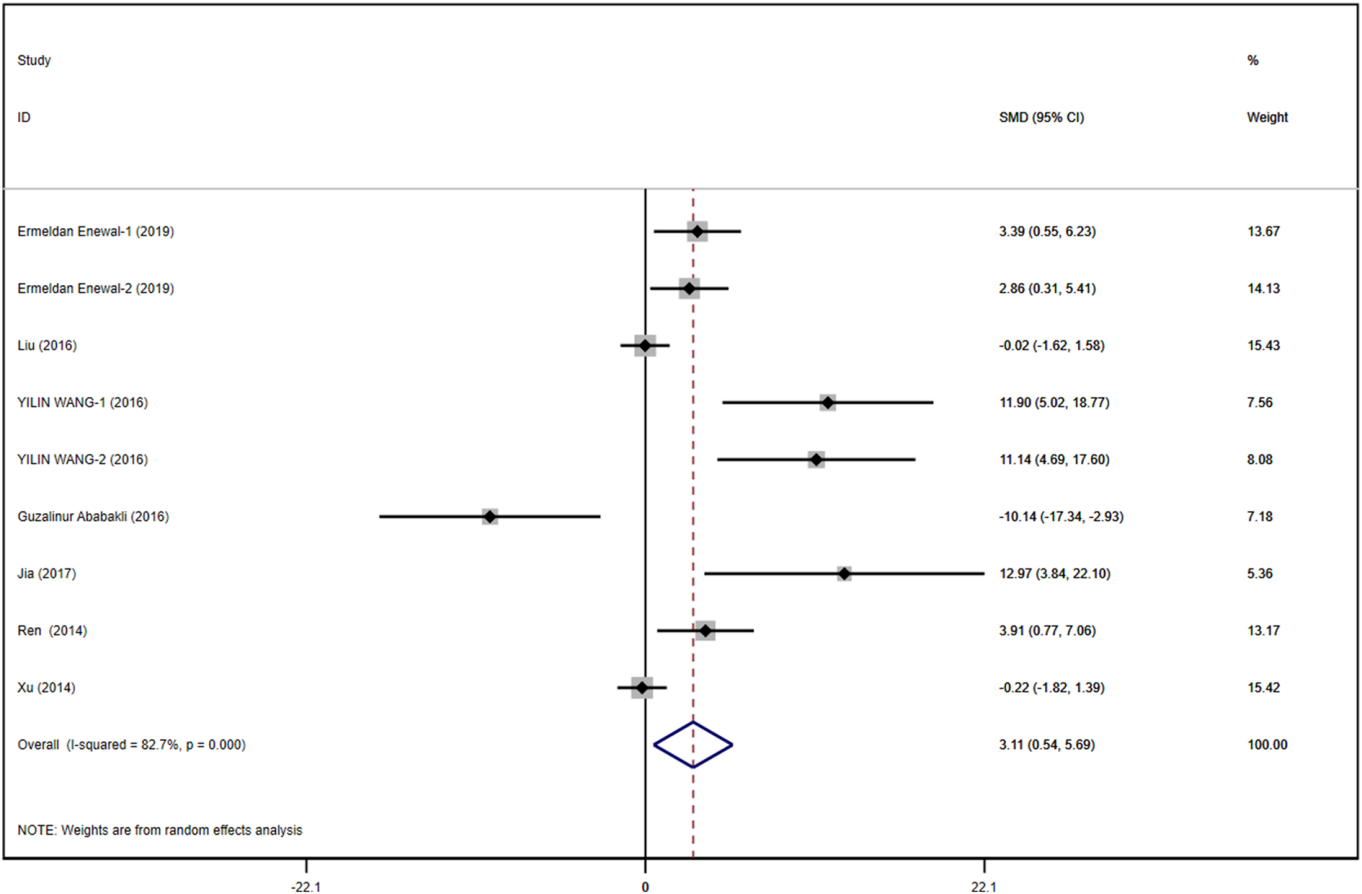
A490

A total of 9 studies with DPSCs samples reported differences in the A490 index between the control group and the TGF-β group. All studies were randomized controlled trials of samples from animals or people. The TGF types included were mainly TGF-β-1 and TGF-β-3. We synthesized the data using Stata 16.1 software. A heterogeneity test was also performed using Stata16.1 software, and it was found that I-square was 82.7%, *P* < 0.001. Therefore, we used SMD in a random effect model as an indicator to evaluate the overall effect and found (SMD 3.11, 95% CI [0.54–5.69]), suggesting that TGF-β promoted the proliferation of dental pulp stem cells. We provide a forest graph in Fig. 3.

##### BSP

**Fig. 4:**
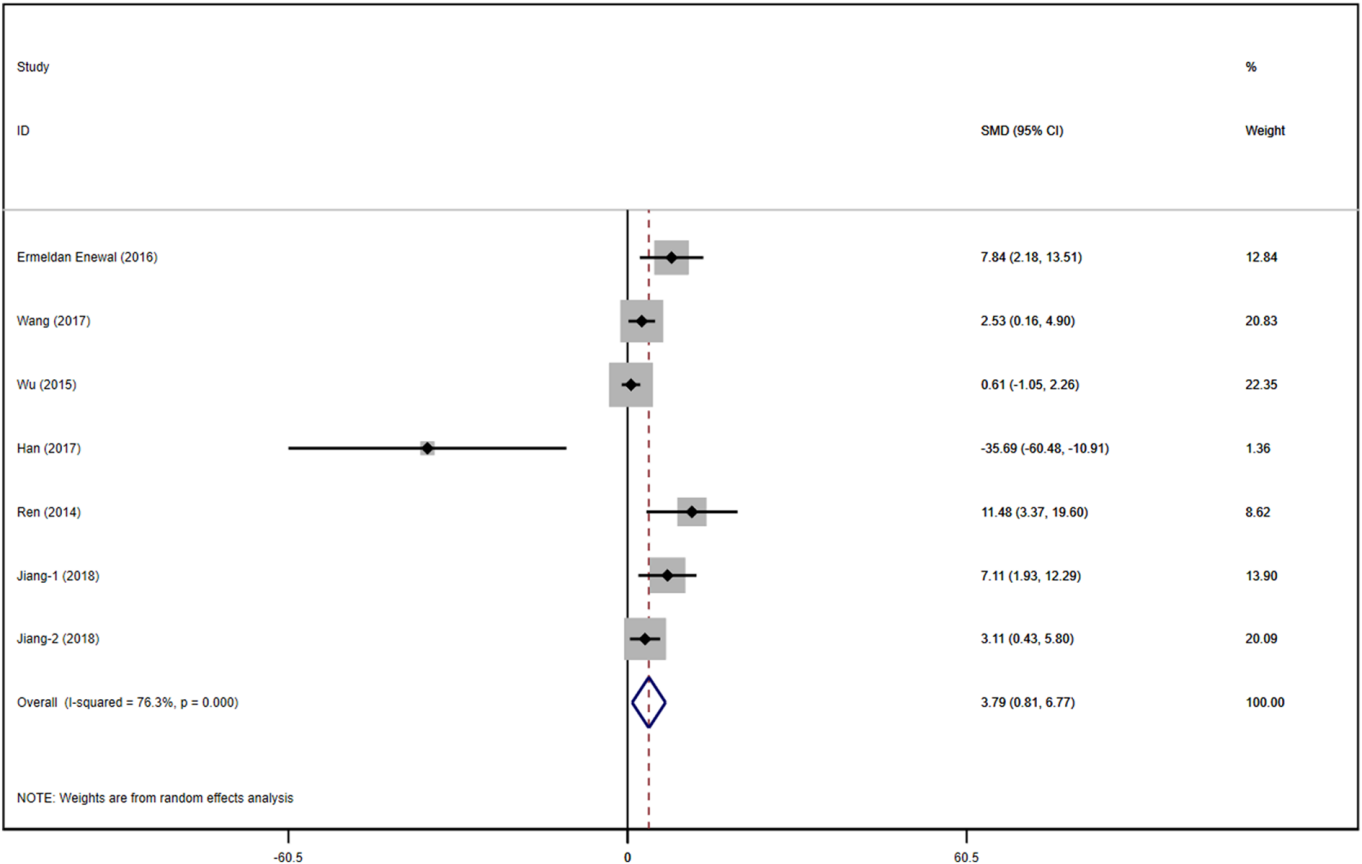
Content of BSP

**Fig. 5:**
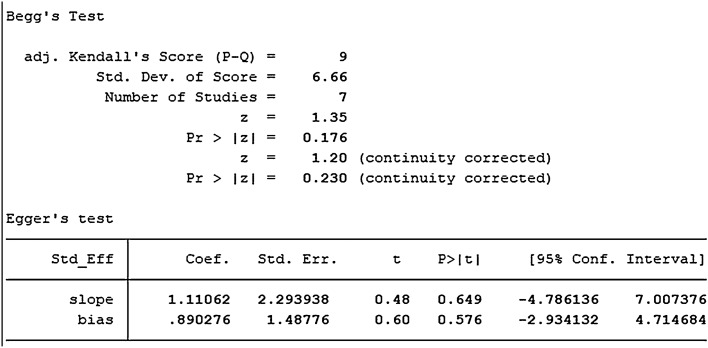
Content of BSP

A total of 7 studies with DPSCs samples reported differences in the content of BSP between the control group and the TGF-β group. All studies were randomized controlled trials of samples from animals or people. The TGF types included were mainly TGF-β-1 and TGF-β-3. Data were summarized using Stata 16.1 software. A heterogeneity test was also performed using Stata16.1 software, and the I-square was equal to 76.3%, *P* < 0.001, which suggests a higher heterogeneity than we set before. Hence, we used SMD in a random effect model as an indicator to evaluate the overall effect and found (SMD 3.11, 95% CI [0.81–6.77]), suggesting that TGF-β promotes the osteogenesis and proliferation of the dental pulp stem cells. We provide a forest graph in Fig. 4 for BSP. We performed Begg's test and egger's test to evaluate the potential publication bias and found no significant publication bias in the studies included in BSP. For more details, see Fig. 5.

#### Col-1

**Fig. 6:**
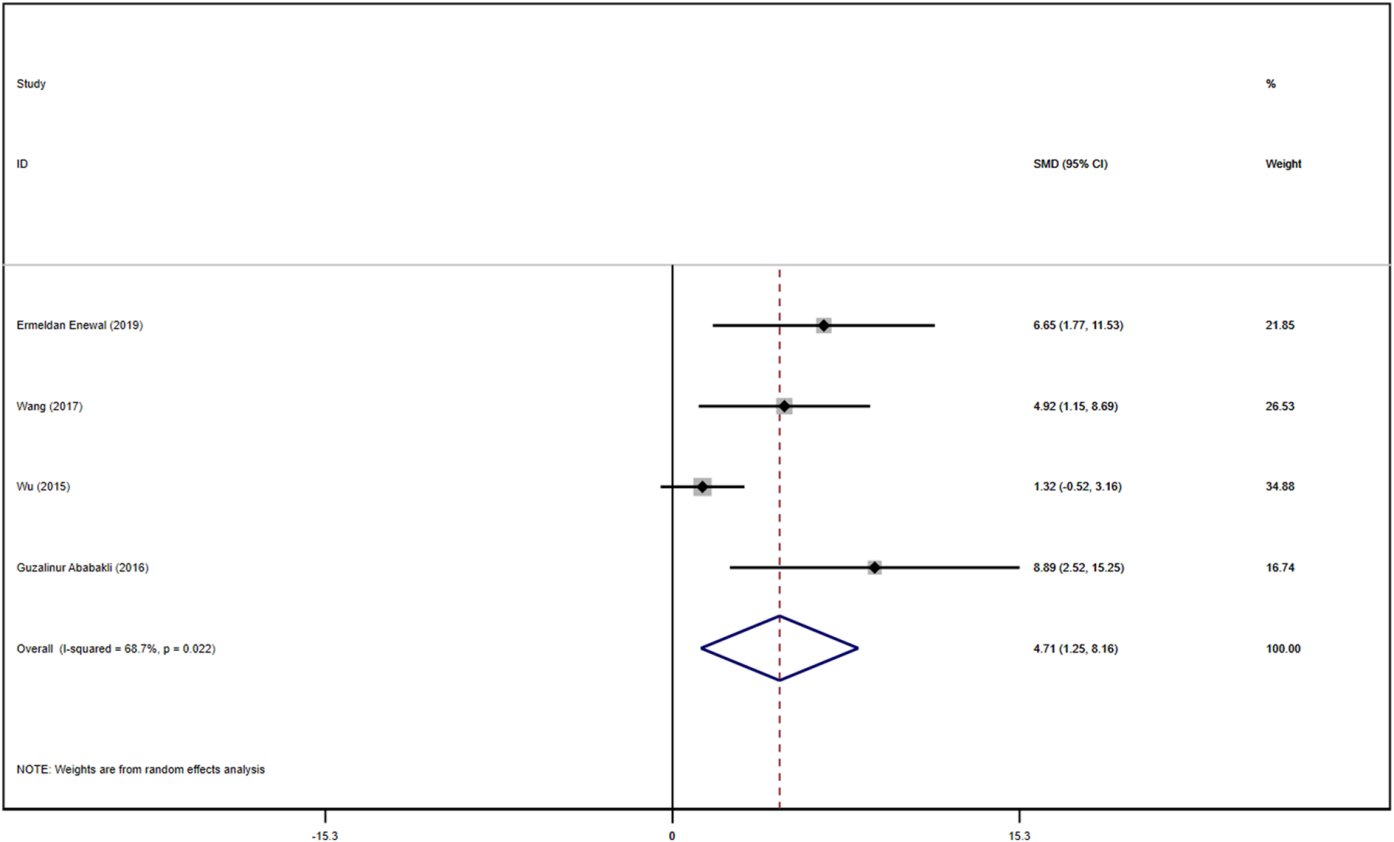
Expression of Col-1

**Fig. 7:**
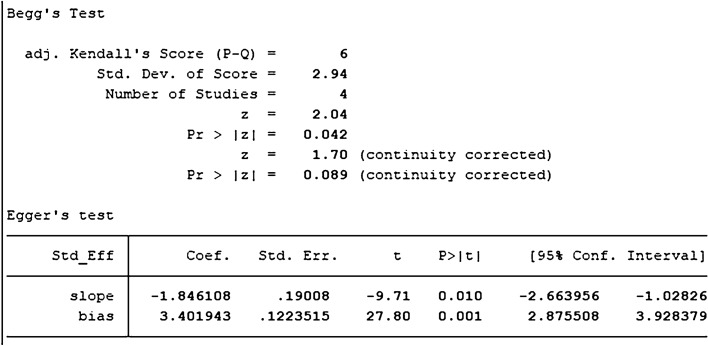
Expression of Col-1

A total of 4 studies with DPSCs samples reported differences in the expression of Col-1 in dental pulp stem cells between the control group and the TGF-β group. All studies were randomized controlled trials of samples from animals or people. The TGF types included were mainly TGF-β-1 and TGF-β-3. Data were summarized using Stata 16.1 software. Heterogeneity test was also performed using Stata16.1 software, and the I-square was equal to 68.7%, *P* < 0.001, which is higher heterogeneity than we set before. The evidence is medium-quality evidence. Moreover, we used SMD in a random effect model as an indicator to evaluate the overall effect and found (SMD 4.71, 95% CI [1.25–8.16]), suggesting that TGF-β promotes the proliferation of dental pulp stem cells. We provide a forest graph in Fig. 6 for the expression of Col-1. We performed Begg's test and egger's test to evaluate the potential publication bias and found no significant publication bias in the studies included in the subgroup of expression of Col-1. For more details, see Fig. 7.

##### OCN

**Fig. 8:**
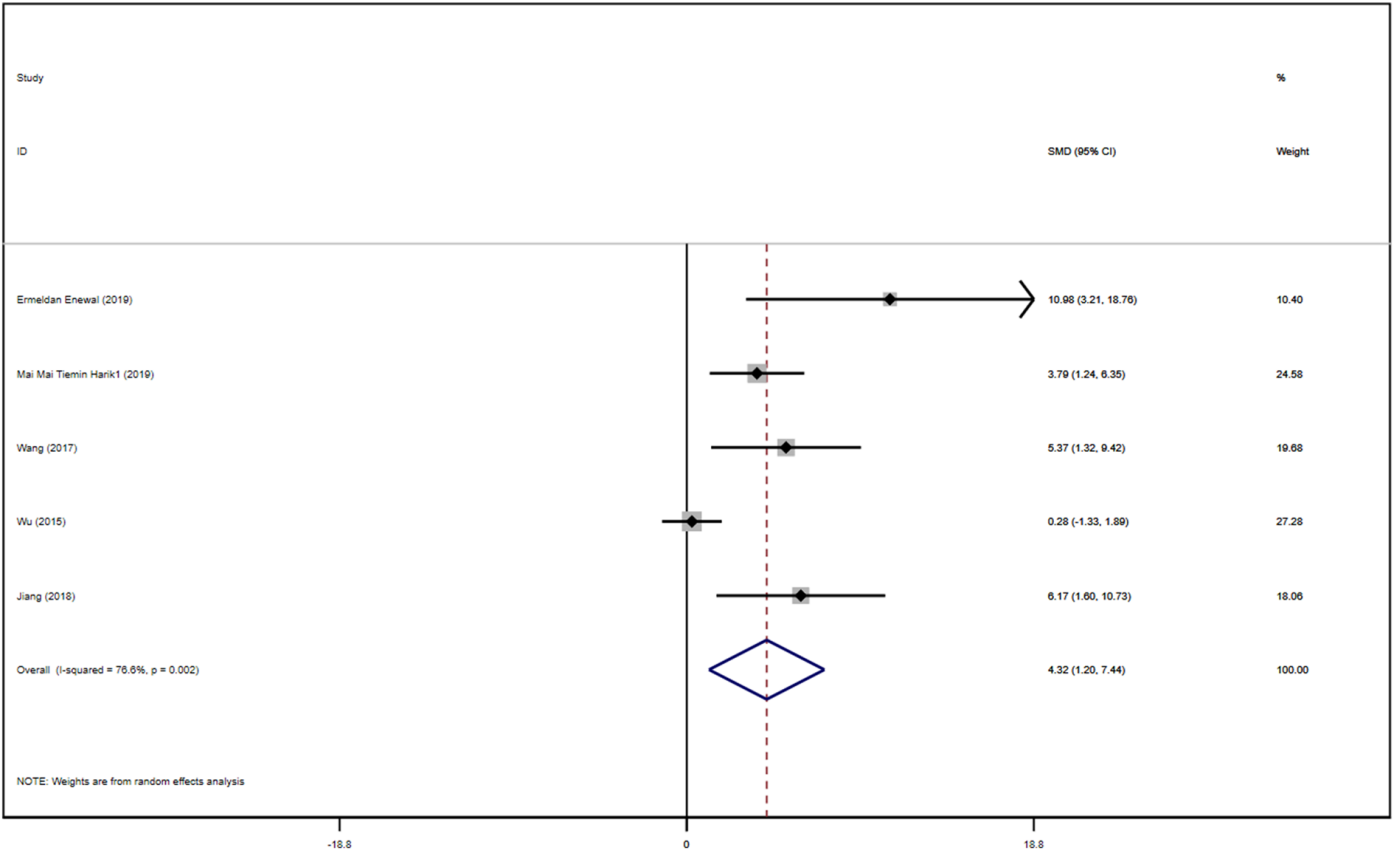
Content of OCN

**Fig. 9:**
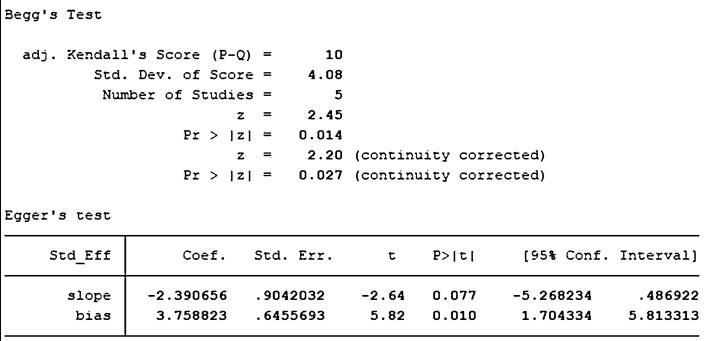
Content of OCN

Five studies with DPSCs samples reported differences in OCN content between the control group and the TGF-β group. The TGF types were mainly TGF-β-1, TGF-β-3, except for TGF-β2. We synthesized the content of OCN using Stata 16.1 software and found the included studies were all randomized controlled trials of samples from animals or people. A heterogeneity test was also performed by Stata16.1 software, and it was found that I-square was 76.6%, *P* = 0.002. Therefore, we used SMD in a random effect model as an indicator to evaluate the overall effect and found (SMD 4.32, 95% CI [1.20–7.44]), suggesting that TGF-β promoted the mineralization and calcification of dental pulp stem cells. We provide a forest graph in Fig. 8. Stata16.1 performed Begg's test and egger's test, and the publication bias was not significant. For more details, see Fig. 9.

#### Runx-2

**Fig. 10:**
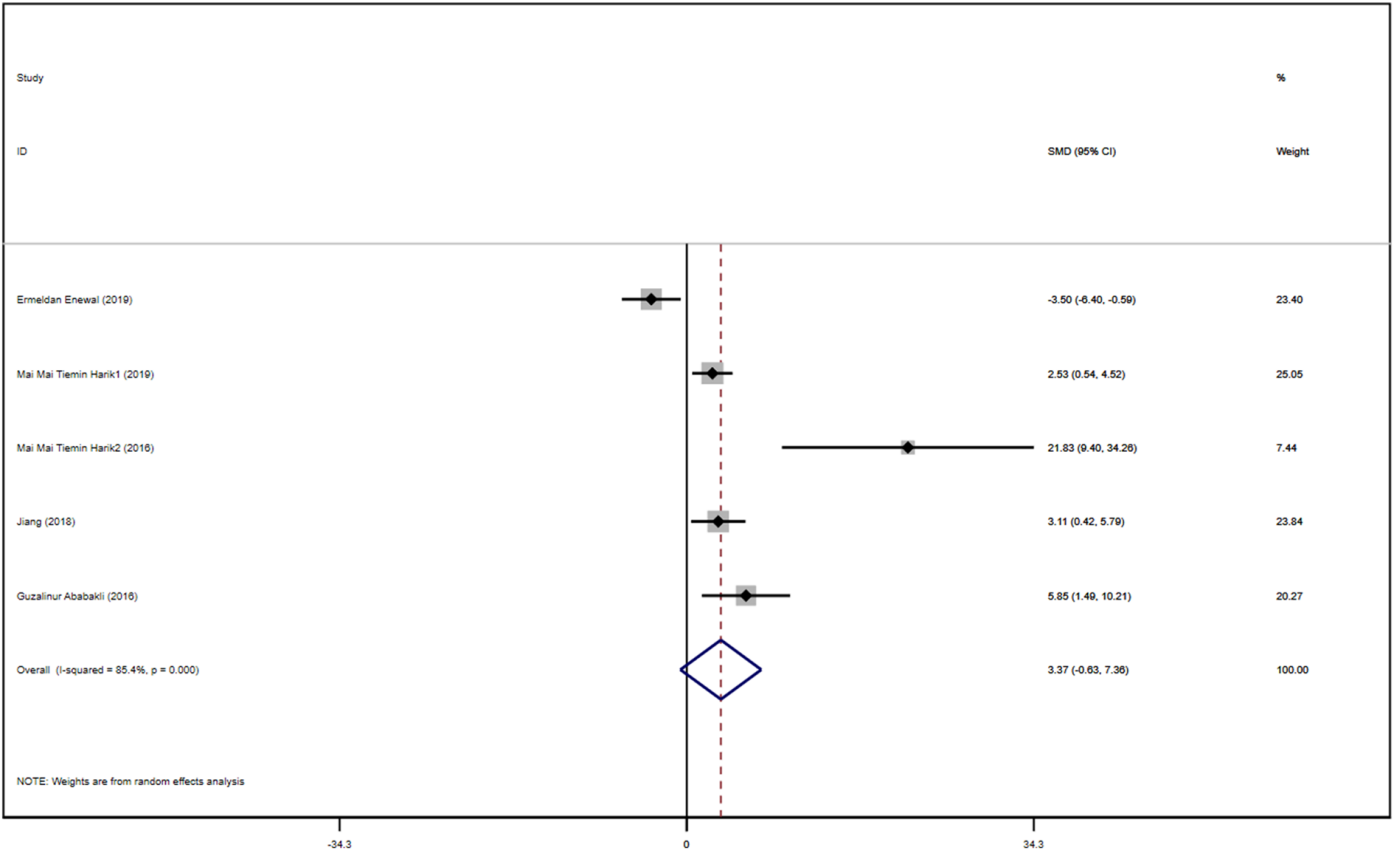
Expression of Runx-2

**Fig. 11:**
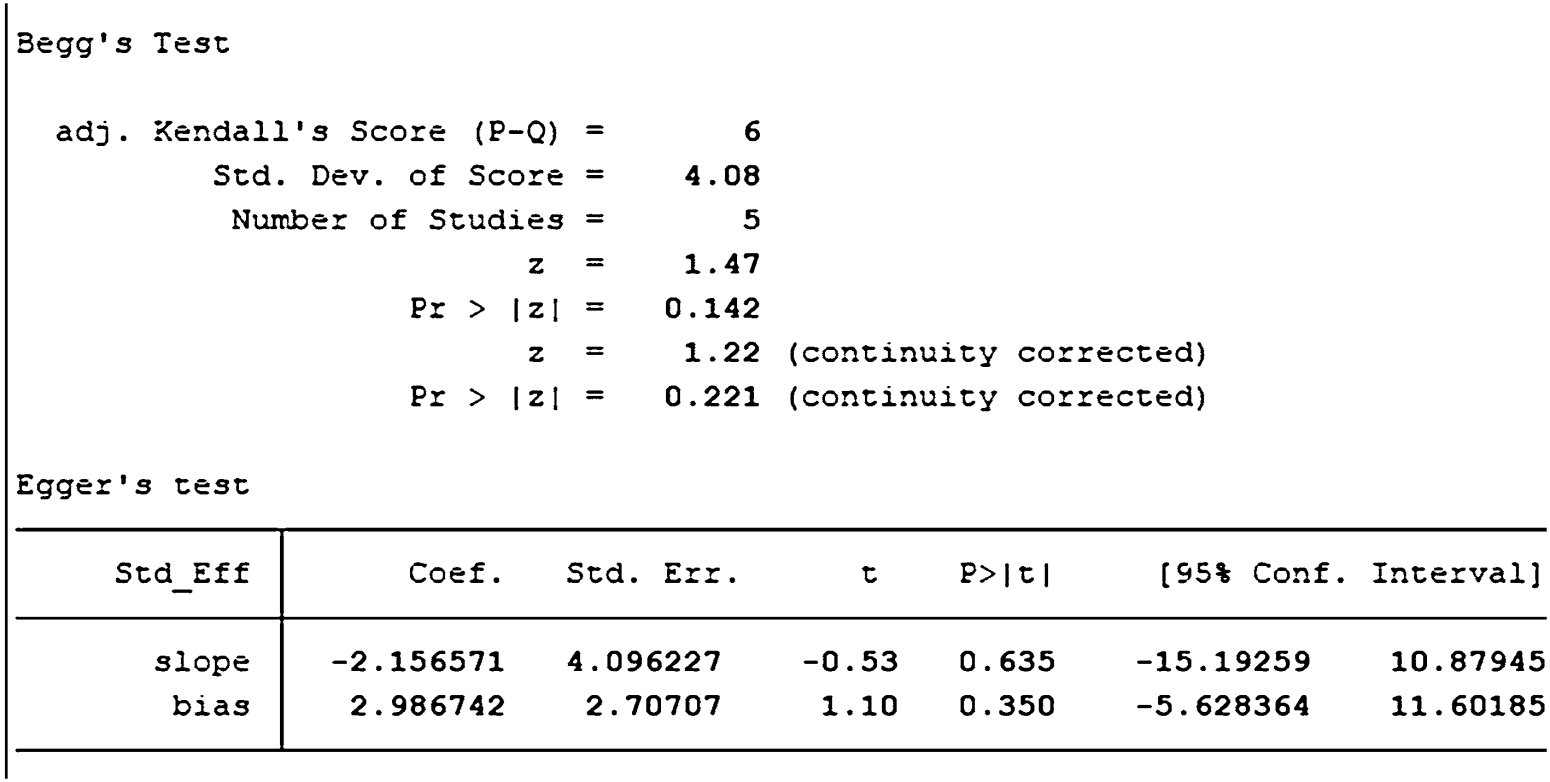
Expression of Runx-2

A total of five studies with DPSCs samples reported differences in the expression of Runx-2 in dental pulp stem cells between the control group and the TGF-β group. All studies were randomized controlled trials of samples from animals or people. The TGF types included were TGF-β-1 and TGF-β-3. The expression size was summarized using Stata 16.1 software. The heterogeneity test was also performed using Stata16.1 software, and the I-square was equal to 85.4%, *P* < 0.001, which is higher heterogeneity than we set before. In addition, the evidence is medium-quality evidence. In addition, we used SMD in a random effect model as an indicator to evaluate the overall effect and found (SMD 3.37, 95% CI [− 0.63 to 7.36]), suggesting that TGF-β promotes the proliferation of dental pulp stem cells. We provide a forest graph in Fig. 10 for the expression of Runx-2. We performed Begg's test and egger's test to evaluate the potential publication bias and found no significant publication bias in the studies included in the subgroup of expression of Runx-2. For more details, see Fig. 11.

#### The mineralized nodules

**Fig. 12:**
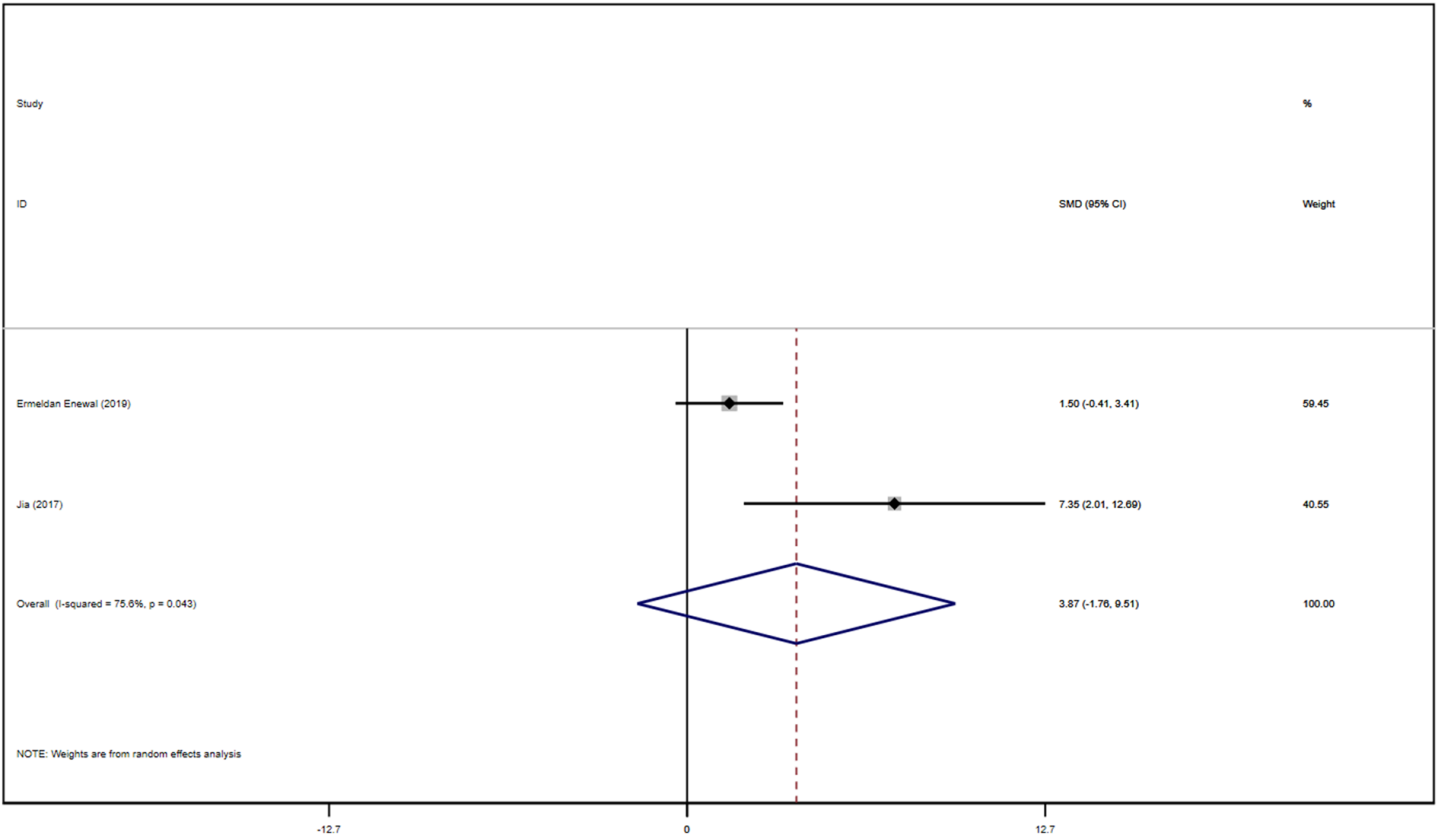
Number of mineralized nodules

**Fig. 13:**
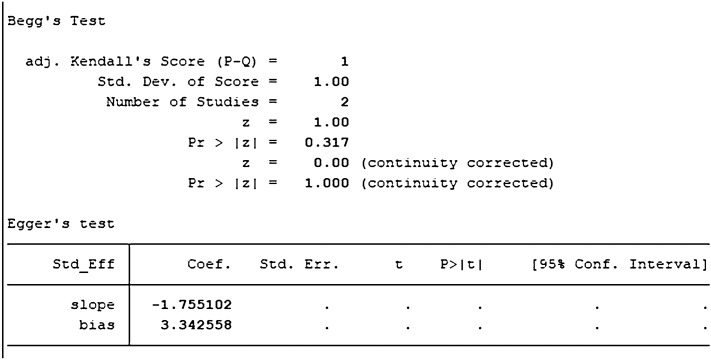
Number of mineralized nodules

Two studies identified with DPSCs samples reported differences in the number of mineralized nodules between control and TGF-β groups. The TGF types were mainly TGF-β-1, TGF-β-3, except for TGF-β2. We synthesized the number of mineralized nodules in all the samples included by Stata 16.1 software and found the included studies were all randomized controlled trials of samples from animals or people. We performed a heterogeneity test by Stata16.1 software, in which we found that the I-square was 75.6%, *P* = 0.043. Therefore, we used SMD in a random effect model as an indicator to evaluate the overall effect and found (SMD 3.87, 95% CI [− 1.76 to 9.51]), suggesting that TGF-β does not increase the number of mineralized nodules in the dental pulp. We provide a forest graph in Fig. 12. Stata16.1 performed Begg's test and egger's test, and the publication bias was not significant. For more details, see Fig. 13.

## Discussion

### Findings

The pooled data showed that TGF-β could promote the proliferation and osteogenesis of dental pulp stem cells. All the outcomes summarized supported this conclusion except for the number of mineralized nodules: TGF-β increased the A490 index, promoted the production of BSP, promoted the expression of Col-1 and Runx-2, increased the content of OCN in dental pulp, and had no significant effect on the number of mineralized nodules in dental pulp stem cells. Samples included in this study were from mammalian and human dental pulps. Mammalian multipotent stem cells and multipotent stem cells have suitable homology and similarity. Hence, we combined the indicators of these samples to obtain a conclusion applicable to mammals and humans. The TGF-β family consists of 33 members, such as TGF-βs, activators, inhibin, and bone morphogenetic protein (BMP). TGF-βs [25] are mainly divided into TGF-β1, TGF-β2, and TGF-β3. Among them, TGF-β1 in TGF-βs has molecular functions, such as regulating cell proliferation, differentiation and wound healing, which has been reported to play an essential role in the pathophysiological processes, such as liver fibrosis [25–28]. However, it needs to be noted that there is a review about the part of TGF-β on articular cartilage. In cartilage formation, TGF-β triggers the aggregation of MSC. After aggregation, TGF-β signaling further stimulates chondrocyte proliferation and inhibits chondrocyte hypertrophy and maturation, which may challenge our conclusion [29]. Human dental pulp stem cells (HDPSCs) are adult stem cells derived from ectoderm and derived migrating neural crest cells. DPSCs can differentiate into odontoblasts, osteoblasts, adipocytes, chondrocytes, muscle cells, and neuronal cells under suitable culture conditions in vitro and in vivo. The ability of dental differentiation is very essential in oral research [30]. It has been reported that osteogenic induction can significantly enhance the mineralization ability of DPSCs, and TGF-β is a potent inducer of osteogenesis [31–33]. However, previous studies have drawn different conclusions on the osteogenic induction of DPSCs by TGF-β, especially on the role of TGF-β in promoting dental pulp mineralization. This review aims to determine the effects of TGF-β on the osteogenesis and proliferation of the dental pulp stem cells and to add new evidence for the physiological effects of TGF-β on mammals and humans.

### Heterogeneity

We believe that heterogeneity is considerable when I-squared is greater than 45%, which is generally lower than the heterogeneity of measures in this study. Furthermore, the possible sources of the heterogeneities were thought to be the following items: 1. We synthesized samples from human and mammalian dental pulps. Although this does not bring problems in ethical and physiological perspectives, it may lead to heterogeneity; 2. Different researchers and laboratories may have significant differences in the observation of sample indicators; 3. The animal weight included in this review was somehow different, which means that data may come from mammals of different ages. The degree of osteogenesis varies with age, leading to the heterogeneity of osteogenesis and mineralization indicators. Moreover, the human samples included in this review come from people of different ages, mainly children and infants.

### Shortcomings

There are shortcomings in this review: 1. The number of studies and samples included about some outcomes is relatively small, leading to the contingency of results. 2. The TGF-β types included in this review were only included TGF-β1 and TGF-β3, while the effect of TGF-β2 on dental pulp stem cells was not discussed. 3. This review only summarized the effects of TGF-β on the osteogenesis and proliferation of dental pulp stem cells from the perspectives of morphology and molecular biology. Relevant physiological and embryological mechanisms cannot be demonstrated by the outcomes of this review, which may require more studies to provide more evidence.

## Supplementary Information

**Additional file 1:** PRISMA Checklist.

## Data Availability

All data generated or analysed during this study are included in this published article.

## Abbreviations

DPSCs: Dental pulp stem cells
CNKI: China national knowledge infrastructure
BSP: Bone sialoprotein
Col-1: Col plasmid-1
OCN: Osteocalcin
Runx-2: Runt-related transcription factor 2
TGF-&beta/TGF-β: Transforming growth factor-&beta;
Smad protein: Sma and Mad proteins
TGF-βR1: TGF-β1 receptor
BMP: Bone morphogenetic protein
HDPSCs: Human dental pulp stem cells
